# Different Predictors of Right and Left Ventricular Metabolism in Healthy Middle-Aged Men

**DOI:** 10.3389/fphys.2015.00389

**Published:** 2015-12-21

**Authors:** Marja A. Heiskanen, Tuija Leskinen, Jari-Joonas Eskelinen, Ilkka H. A. Heinonen, Eliisa Löyttyniemi, Kirsi Virtanen, Jussi P. Pärkkä, Jarna C. Hannukainen, Kari K. Kalliokoski

**Affiliations:** ^1^Turku PET Centre, University of TurkuTurku, Finland; ^2^School of Sport Science, Exercise and Health, University of Western AustraliaCrawley, WA, Australia; ^3^Department of Biostatistics, University of TurkuTurku, Finland

**Keywords:** right ventricle, left ventricle, metabolism, glucose uptake, free fatty acid uptake, positron emission tomography, magnetic resonance imaging, exercise

## Abstract

Dysfunction of the right ventricle (RV) plays a crucial role in the outcome of various cardiovascular diseases. Previous studies on RV metabolism are sparse although evidence implies it may differ from left ventricular (LV) metabolism. Therefore, the aims of this study were (1) to determine predictors of RV glucose uptake (GU) and free fatty acid uptake (FFAU) and (2) to compare them to predictors of LV metabolism in healthy middle-aged men. Altogether 28 healthy, sedentary, middle-aged (40–55 years) men were studied. Insulin-stimulated GU and fasting FFAU were measured by positron emission tomography and RV and LV structural and functional parameters by cardiac magnetic resonance. Several parameters related to whole-body health were also measured. Predictors of RV and LV metabolism were determined by pairwise correlation analysis, lasso regression models, and variable clustering using heatmap. RVGU was most strongly predicted by age and moderately by RV ejection fraction (EF). The strongest determinants of RVFFAU were exercise capacity (peak oxygen uptake), resting heart rate, LVEF, and whole-body insulin-stimulated glucose uptake rate. When considering LV metabolism, age and RVEF were associated also with LVGU. In addition, LVGU was strongly, and negatively, influenced by whole-body insulin-stimulated glucose uptake rate. LVFFAU was predicted only by LVEF. This study shows that while RV and LV metabolism have shared characteristics, they also have unique properties. Age of the subject should be taken into account when measuring myocardial glucose utilization. Ejection fraction is related to myocardial metabolism, and even so that RVEF may be more closely related to GU of both ventricles and LVEF to FFAU of both ventricles, a finding supporting the ventricular interdependence. However, only RV fatty acid utilization associates with exercise capacity so that better physical fitness in a relatively sedentary population is related with decreased RV fat metabolism. To conclude, this study highlights the need for further study designed specifically on less-known RV, as the results on LV metabolism and physiology may not be directly applicable to the RV.

## Introduction

While the left ventricle (LV) has been studied extensively, the right ventricle (RV) has long remained understudied and regarded as a “passive bystander” providing merely the pulmonary circulation (Voelkel et al., 2006; Haddad et al., 2008). As the two ventricles are connected in series, failure in either of the ventricles decreases cardiac output and can ultimately lead to premature death. RV failure is a strong determinant of survival in patients with pulmonary arterial hypertension (PAH), and its role in the clinical outcome of various cardiovascular diseases has been recognized (Zornoff et al., 2002; Voelkel et al., 2006; Haddad et al., 2008; Walker and Buttrick, 2013). Cardiac metabolism and contraction are closely integrated because without sufficient fuel the heart is not able to fulfill the circulatory demands. Despite of the clinically significant role of RV, studies on its basic metabolism even in healthy subjects are scarce.

As the energy reserves of the heart are small, cardiac function is closely tied to its capability to efficiently utilize different substrates in order to generate ATP. Normal healthy heart produces almost all (>95%) of its ATP through mitochondrial oxidative phosphorylation and the remaining part through glycolysis (Stanley et al., 2005; Lopaschuk and Kelly, 2008). Heart is also an “omnivore,” producing most of its energy for mitochondrial metabolism from fatty acids (60–90%) and the remaining 10–40% from carbohydrates such as glucose and lactate (Bing et al., 1953; van der Vusse et al., 2000). The contribution of different energy substrates to oxidative ATP production depends on various conditions such as alterations in plasma substrate supply, hormonal control, workload, and oxygen supply to the heart (Lopaschuk and Kelly, 2008; Peterson and Gropler, 2010). Alterations of substrate utilization can be acute adaptations to changes in physiological condition, or chronic changes caused for example by aging, obesity, or disease (Peterson and Gropler, 2010). For instance, RV glucose uptake has been shown to be increased along with decreases in RV function, such as ejection fraction (EF), in patients with PAH (Oikawa et al., 2005; Can et al., 2011; Lundgrin et al., 2013; Yang et al., 2014) or heart failure (Mielniczuk et al., 2011). However, the predictors of RV metabolism in healthy heart are poorly understood.

Therefore, the two main aims of this study were to determine (1) predictors of RV metabolism and (2) compare them to predictors of LV metabolism in healthy middle-aged men. Cardiac energy substrate metabolism was measured by determining insulin-stimulated RV and LV glucose uptake (GU) and fasting free fatty acid uptake (FFAU) using positron emission tomography (PET). Furthermore, cardiac magnetic resonance (CMR), regarded as the gold standard for cardiac structural assessment (Grothues et al., 2004), was performed to measure RV and LV structure and function. Several parameters related to whole-body health were also measured. Although previous studies on RV metabolism are sparse, evidence implies that it may differ from that of the LV as the early divergence in the embryologic origins of the RV and LV myocardium may result in the chamber-specific responses (Zaffran et al., 2004; Adrogue et al., 2005; Walker and Buttrick, 2013; Sankaralingam and Lopaschuk, 2015). We therefore hypothesized that the predictors of RV and LV metabolism may be different.

## Methods

### Subjects

The participants were recruited with advertisements in local newspapers, through personal contacts, and using electronic and traditional bulletin boards. Before the study, subjects were interviewed and thoroughly examined by a medical doctor. A candidate was accepted to the study if following criteria were fulfilled: male sex, age 40–55 years, body mass index 18.5–30 kg·m^−2^, and no exercise on regular basis (twice a week or less, VO_2peak_ ≤ 40 ml·kg^−1^·min^−1^). A candidate was excluded if he had a condition which could potentially endanger subject's health during the study or interfere with the interpretation of the results (high blood pressure >140/90 mmHg, chronic disease or medical defect requiring medical treatment, eating disorders, use of steroids, narcotics, tobacco, or other substrates, heavy use of alcohol). Finally, 28 participants fulfilled the criteria. The study was conducted according to Declaration of Helsinki, and the study protocol was approved by the ethical committee of the Hospital District of Southwest Finland, Turku (decision 95/180/2010§228). Written informed consent was obtained from all subjects before the beginning of the study.

### Measurements

#### PET measurements

Before the PET experiments, antecubital veins from both arms were cannulated, one for the administration of PET tracers and other infusions (glucose-insulin clamp, only in FDG-PET study) and the opposite one for blood sampling during the study. The arm used for blood sampling was heated with an electrically powered cushion for the whole duration of the study to “arterialize” the venous blood. The subject was positioned into the PET scanner in a supine position with the thoracic region in the scanning area of the gantry. The PET imaging was performed with GE Advance PET/CT scanner (General Electric Medical System, Milwaukee, WI, USA).

FFAU was studied using 14(*R,S*)-(18F)fluoro-6-thia-heptadecanoic acid (18F-FTHA) as a tracer, which is also shown to trace the free fatty acid beta-oxidation in the heart (Takala et al., 2002). Once the tracer [155 (SD 9) MBq] was injected into the vein, the scanning was started immediately and continued for 40 min in 4 × 15 s, 6 × 20 s, 2 × 60 s, 2 × 150 s, and 6 × 300 s time frames. Blood samples for plasma radioactivity determination (Wizard 1480 3; Wallac, Turku, Finland) and the calculation of plasma input function were collected at 4, 5, 7.5, 10, 20, 30, and 40 min after the injection of the tracer. The activities of the left ventricle chamber during the first 3 min (first 10 frames) were used to determine the early part of the tissue input function. As FTHA is metabolized (Takala et al., 2002), blood samples were analyzed at time points 5, 10, 20, 30, and 40 min after the tracer injection to determine FTHA metabolites and to correct the input function for pure plasma FTHA input function. Free fatty acid concentrations were measured before FTHA-PET study and 20 min after the start of the study to calculate the final FFAU values.

On a separate day, GU was measured using 2-deoxy-2-(18F)fluoro-D-glucose (18F-FDG). The tracer [157 (SD 10) MBq] was injected into the vein and scanning was performed in similar time frames as in the FTHA study. The myocardial GU scanning was performed during euclycemic hyperinsulinemic clamp at fasted state (explained in detail later in the text). FDG was injected 90 (SD 16) min after the start of the euclycemic hyperinsulinemic clamp when the subject had reached the stable glucose concentrations at the level of 5 mmol·l^−1^ (±0.5). Except for the clamp, FDG-PET was performed similarly to FTHA-PET. As FDG is not metabolized in the body (Rudroff et al., 2015), no additional blood samples were needed for metabolite correction. Glucose concentrations were measured at every 5 min and the whole period was used for calculations of the final GU values.

#### PET image analysis

**Figure 1:**
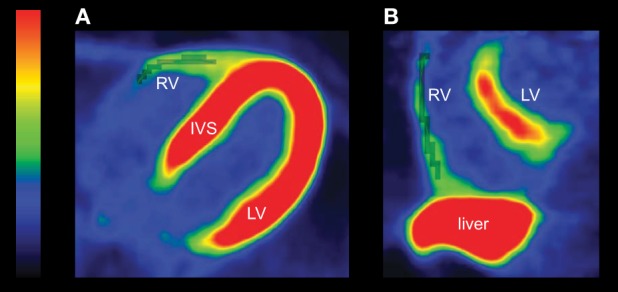
**(A)** An example of FDG-PET image in one transaxial slice. The RV ROIs are drawn to the entire RV free wall (gray shaded voxels). The U-shaped left ventricle is clearly seen. **(B)** An example of FTHA-PET image in one sagittal slice. FTHA activity of the liver is high, and it overlaps with the RV. Therefore, the lower border of the RV is not included in the ROI (gray shaded voxels) to avoid cross-contamination of the signal between the tissues. Color bar shows the tracer uptake rate with red and yellow indicating the highest uptake. RV, right ventricle; LV, left ventricle; IVS, interventricular septum.

PET image raw files were corrected for attenuation, dead time, and decay. Images were reconstructed using 3D-OSEM procedure and analyzed using Carimas software (version 2.9, www.turkupetcentre.fi/carimas). In FDG-PET images, regions of interest (ROIs) were drawn manually to the entire RV free wall in transaxial planes (Figure 1A). For LV, both the interventricular septum and the entire LV free wall were included in ROIs. In FTHA-PET images, ROIs were drawn to entire RV free wall except for the lower border of RV to avoid spillover due to the high tracer uptake in liver (Figure 1B). For LV, ROIs for FTHA-PET images were drawn similarly as in FDG-PET. Once the ROIs were manually defined, the tissue time activity curves were extracted from dynamic PET data. Fractional tracer uptake rate was calculated from tissue and plasma time activity curves by using graphical analysis (Patlak et al., 1983). The rate of GU and FFAU was calculated by multiplying the fractional tracer uptake rate with the plasma glucose and FFA concentration during the scanning, respectively.

#### CMR measurements

RV and LV structure and function was assessed by CMR using a Philips 1.5T Gyroscan Intera CV Nova Dual MR scanner (Philips Medical Systems, Best, The Netherlands). With the subject in supine position, cardiac long axis, and short axis planes were planned from localizer images. The images over the entire cardiac cycle was obtained with cardiac cine sequencing: steady-state free-precession cine image series were acquired with balanced turbo field echo pulse sequence to have a stack of 10–14 parallel slices in short axis plane and 4 slices in four-chamber plane. The repetition time was 3.4 ms, echo time 1.7 ms, flip angle 60°, acquisition matrix 192 × 192, reconstruction matrix 256 × 256, field of view 320–360 nm, slice thickness 6 mm without gap between slices, phases per cardiac cycle 25, and two slices per breath-hold resulting to breath holds of 10–18 s.

#### CMR image analysis

**Figure 2:**
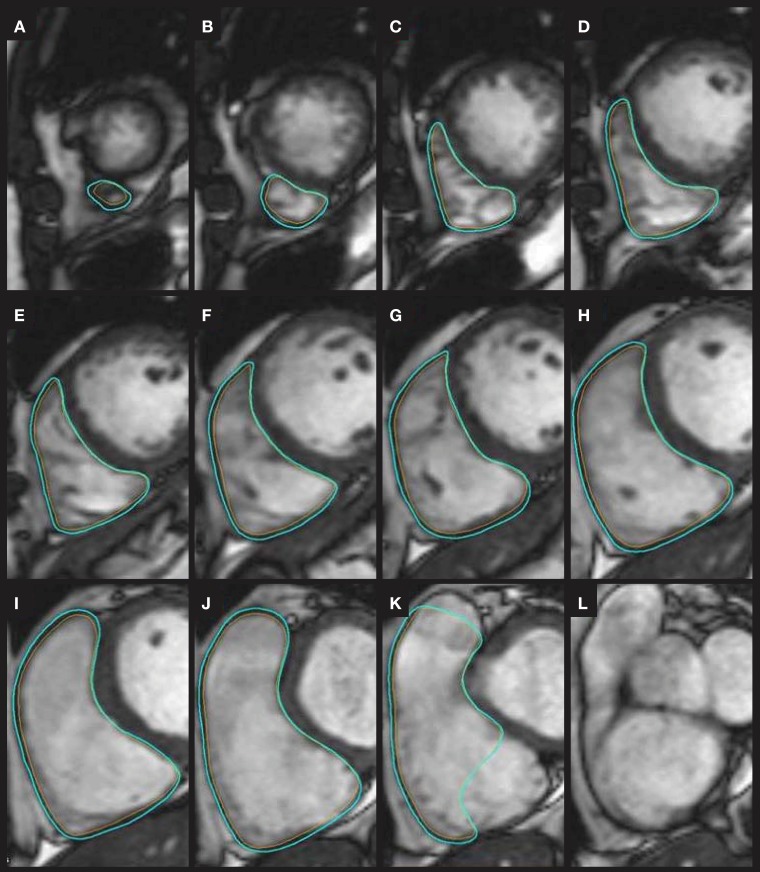
**An example of tracing the endocontours (orange) and epicontours (light blue) on the RV end-diastolic CMR images from the most apical slice (A) through slices (B–J) to the most basal slice (K)**. At the most basal slice **(K)**, the pulmonary valve plane and tricuspid valve plane are excluded according to instructions by Prakken et al. (2008). As the trabeculations next to the tricuspid valve plane in slice **(L)** was not visible for the required three phases (figures not shown), no contours were traced to the slice **(L)** anymore.

Image analysis was performed with Philips Extended MR WorkPlace version 2.6.3.5 (Philips Medical Systems, Best, The Netherlands). The short axis cine images were visually inspected to identify the end-systolic and end-diastolic phases with the smallest and the largest ventricular cavities, respectively. Endocardial contours were traced manually on each slice in both end-systole and end-diastole according to the established procedure to overcome the difficulties faced when tracing the most basal slices especially in RV (Prakken et al., 2008). Briefly, the contour was traced on the most basal RV end-diastolic slice only if the trabeculations stayed visible for a minimum of three following phases. For determination of the RV mass, epicardial contours were traced in end-diastolic slices so that epicardial border overlapped with endocardial border at valve planes and at the septum. Hence, RV mass corresponds to the free wall mass and does not include the septum and trabeculations (Figure 2). For LV, the endocardial contours excluded papillary muscles and epicontours included interventricular septum (Prakken et al., 2008). Once the endo-and epi-contours were traced, end-diastolic volume (EDV), end-systolic volume (ESV), and mass, along with functional parameters including ejection fraction (EF), and stroke volume (SV) were automatically calculated by the image analysis software for both RV and LV.

#### Euclycemic hyperinsulinemic clamp and M-value

The insulin clamp was performed according to original description by DeFronzo et al. (1979) after the subjects had fasted for at least 10 h. A primed-constant insulin (Actrapid 100 U · mL^−1^, Novo Nordisk, Bagsvaerd, Denmark) infusion was started with the rate of 40 mU per m^2^ of body surface area in minute during the first 4 min. Then, the infusion rate was reduced to 20 mU per m^2^ in minute for the time interval 4–7 min. After 7 min, the infusion rate was further reduced to 10 mU per m^2^ in minute for the rest of the clamp. Exogenous glucose infusion was started 4 min after the start of the insulin infusion with a rate of [subjects weight (kg) · 0.1]g · h^−1^. At the 10-min time point, glucose infusion was doubled and after that further adjusted according to blood glucose concentration aiming at the steady level of 5 mmol · l^−1^. Arterialized venous blood samples were collected before the clamp and every 5 min during the first 30 min of the clamp to determine the glucose concentration for adjusting the glucose infusion rate. After 30 min, blood samples were taken at every 10 min and modification to the infusion rate were made when necessary. Whole-body insulin stimulated glucose uptake rate (*M*-value) was calculated from the glucose values obtained in the steady state. Thus, *M*-value is a measure of whole-body glucose uptake, and it is considered as the most definitive measure of insulin sensitivity in humans (DeFronzo et al., 1979).

#### Maximal exercise test (VO_2peak_)

The subjects performed a maximal exercise test on a cycle ergometer (Ergoline 800s; VIASYS Healthcare, Germany). The test started at 50 W and the load was increased by 30 W at every 2 min until exhaustion. Ventilation and gas exchange was measured (Jaeger Oxycon Pro; VIASYS Healthcare) and reported as the mean value per minute. The peak respiratory exchange ratio was ≥ 1.15 and peak blood lactate concentration immediately and after 1 min was ≥ 8.0 mmol·l^−1^ for all subjects. Also, peak heart rate was within 10 beats of the reference value (220–age) for all except one subject. The highest 1-min mean value of oxygen consumption was defined as VO_2peak_.

#### Body composition

Body composition was measured using a bioimpedance monitor (InBody 720, Mega Electronics Ltd., Kuopio, Finland).

### Statistical methods

All demographic and myocardial data in Tables 1, 2 are presented as mean and 95% confidence intervals. Normal distribution of the variables was tested using Shapiro-Wilk test. Due to non-normal distribution in RVESV and RVEDV, logarithmic transformations were done to these variables to achieve normal distribution.

**Table 1:** **Demographic data and whole-body findings for 28 subjects**.

	**Mean**	**95% Confidence interval**
Age (years)	48	(45, 50)
Height (cm)	179	(177, 181)
Weight (kg)	83.6	(80.2, 87.0)
BMI (kg·m^−2^)	26.1	(25.2, 27.1)
Fat%	22.6	(20.9, 24.2)
HR rest	61	(58, 63)
VO_2peak_ (ml·kg^−1^·min^−1^)	34.2	(32.6, 35.8)
*M*-value (μmol kg^−1^·min^−1^) (*n* = 26)	35.3	(29.5, 41.2)

*BMI, body mass index; HR rest, heart rate at rest (mean of screening day and two PET scanning days); VO_2peak_, peak oxygen consumption during the exercise test; M-value, whole-body glucose consumption during glucose-insulin clamp*.

**Table 2:** **Ventricular parameters for RV and LV**.

	**Right ventricle**			**Left ventricle**		
	***n***	**Mean**	**95% CI**	***n***	**Mean**	**95% CI**
GU (μmol·100g^−1^·min^−1^)	25	12.1	(10.7, 13.6)	25	46.3	(40.5, 52.1)
FFAU (μmol·100g^−1^·min^−1^)	24	1.71	(1.50, 1.92)	24	4.22	(3.64, 4.80)
EDV (ml)	27	192	(182, 202)	27	163	(155, 171)
ESV (ml)	27	90.0	(82.9, 97.7)	27	58.0	(53.5, 62.6)
Mass (g)	27	31.8	(30.0, 33.5)	27	122	(115, 130)
EF (%)	27	52.9	(51.0, 54.8)	27	64.6	(63.0, 66.2)
SV (ml)	27	102	(97.5, 106)	27	105	(101, 109)

*CI, confidence interval; GU, glucose uptake; FFAU, free fatty acid uptake; EDV, end-diastolic volume; ESV, end-systolic volume; EF, ejection fraction; SV, stroke volume*.

**Figure 3:**
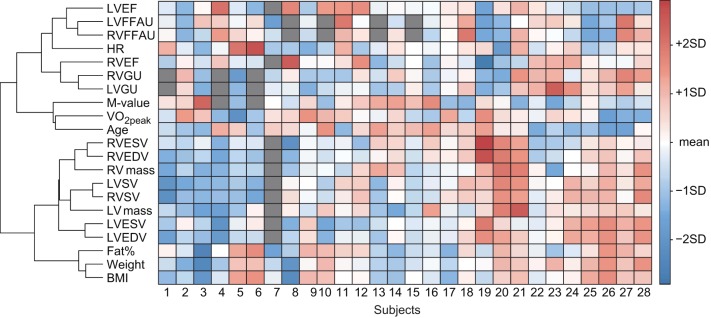
**A visual presentation of the dataset where each matrix element describes a value for a respective variable (columns) and subject (rows)**. Each variable is scaled to zero mean and unit variance. Hence, elements with light colors indicate values close to mean of the given variable, whereas values larger than mean are presented as red and values smaller than mean as blue matrix elements. The tree on the left side of the matrix describes how variables are related to each other according to cluster analysis. The shorter the branches are, the stronger the association between the variables is. For example, RVSV and LVSV are tightly related, as expected. On the other hand, VO_2peak_, age, and *M*-value are clustered together but since the branches are longer, the association is not that strong. Dark gray matrix elements indicate missing values.

Pairwise correlations are reported as Pearson's product-moment correlation coefficients (r). Heatmap (Figure 3) was produced using heatmap.2 function in “gplots” R-package with manhattan distance function and Ward's method for the agglomeration of the clusters. All variables were centered to zero mean and scaled to unit variance. Both rows (variables) and columns (individual subjects) were allowed to be reordered so that similar variable and individual profiles are shown close to each other.

Metabolic parameters (RVGU, RVFFAU, LVGU, LVFFAU) were modeled using lasso regression (Table 3). The **l**east **a**bsolute **s**hrinkage and **s**election **o**perator, or *lasso*, is a method for estimation in linear models introduced by Tibshirani (1996). Lasso minimizes the sum of squared errors as in typical linear regression, but in addition it penalizes for the sum of absolute values of the coefficients. Formally, estimates for coefficients $\hat{\beta }$ are achieved by

$$\hat{\beta }\;=\;{\text{argmin}}_{\beta }\left({\left\|y\;-\;X\beta \right\|}_{2}\;+\;\lambda {\left\|\beta \right\|}_{1}\right),$$

where the first term presents the loss function as in normal linear regression and the second term stands for penalty, whose strength is controlled by parameter λ. Because of the penalty term, some of the coefficients are reduced to zero. The main advantage of lasso is that it simultaneously performs predictor selection and produces an interpretable regression model. Hence, in a final lasso regression model only the predictors having the strongest effects remain non-zero. Conventionally, the non-zero coefficients are considered to be the significant predictors for the model. Since a lasso estimate is a non-linear and non-differentiable function even for a fixed value of lambda, it is difficult to calculate accurate estimate for its standard error or its *p*-value (Tibshirani, 1996). In the original work, standard error estimates are obtained by bootstrap resampling. While a test statistic based on lasso fitted values is proposed (Lockhart et al., 2014), its R-package “covTest” is described as experimental.

**Table 3:** **Lasso regression models for GU and FFAU of both ventricles**.

**Lasso regression model**	***n***	**RMSE**
RVGU = 14.0 − 0.079 · age + 0.025 · RVEF + 0.007· RVSV	24	3.24
LVGU = 80.4 − 0.757 · age − 0.305 ·*M*-value + 0.241· RVEF	24	9.78
RVFFAU = −1.20 + 0.036 · LVEF + 0.019 · HR − 0.012· VO_2peak_− 0.003 ·*M*-value	21	0.36
LVFFAU = 4.65 + 0.022 · LVEF − 0.398 · log(RVESV)	21	1.27

*n, number of subjects used in the model; RMSE, root-mean-square error of the residuals*.

In the present study, lasso modeling was performed using function glmnet in “glmnet” R-package with parameter alpha set to 1. The optimal value for penalization coefficient lambda was determined using repeated cross-validations. The subjects with missing values (due to technical problems) were excluded from the lasso models. The datasets used in the lasso models are relatively small (*n* = 24 for GU and *n* = 21 for FFAU). Obtaining the bootstrap estimates would also require a new cross-validation procedure in order to find optimal λ for each sample set. Because of the small study population, such empirical *p*-values for the coefficient estimates are not determined in the present work. Therefore, the regression coefficients obtained in this work should be interpreted with caution as further study with larger population is required to verify the results. However, the strength of the lasso regression over simple pairwise correlations is that interactions between all the variables are taken into account simultaneously.

All statistical tests were performed as two-sided with statistical significance level set at 0.05. The analyses were performed using R version 3.1.3, the R Foundation for Statistical Computing (http://www.R-project.org/).

## Results

### Baseline statistics

Subject characteristics and whole-body findings are summarized in Table 1 and parameters describing right and left ventricular metabolism, structure and function are presented in Table 2. On average, RV mass and RVGU were roughly 25% of those in LV. In contrast, FFAU in RV was not so much lower and was 40% of the FFAU in LV. Both EDV and ESV were somewhat higher in the RV than in LV, leading to smaller RVEF compared to LVEF. On the other hand, SV was comparable between LV and RV, as expected.

### Individual values

Individual values for every measured parameter are visualized in the heatmap where variables with similar values are clustered together (Figure 3). As expected, RVGU and LVGU as well as RVFFAU and LVFFAU are closely related. Interestingly, RVEF is more closely related to GU whereas LVEF and heart rate associate with FFAU of both ventricles. Age, VO_2peak_, and *M*-value form their own cluster, which is associated with the cluster of myocardial metabolism and ejection fractions. On the other hand, all the other parameters related to myocardial structure and function remain in their own cluster. Weight, BMI, and fat percent seem to be more closely related to the size of the heart rather than its metabolism. For instance, subjects with higher BMI and fat percent often have large-sized heart while their VO_2peak_ and *M*-value are decreased. However, individual profiles vary a lot from subject to subject as can be seen especially in the middle part of the heatmap where no clear pattern for the individual parameter profiles emerges.

### Pairwise correlations (GU and FFAU)

**Figure 4:**
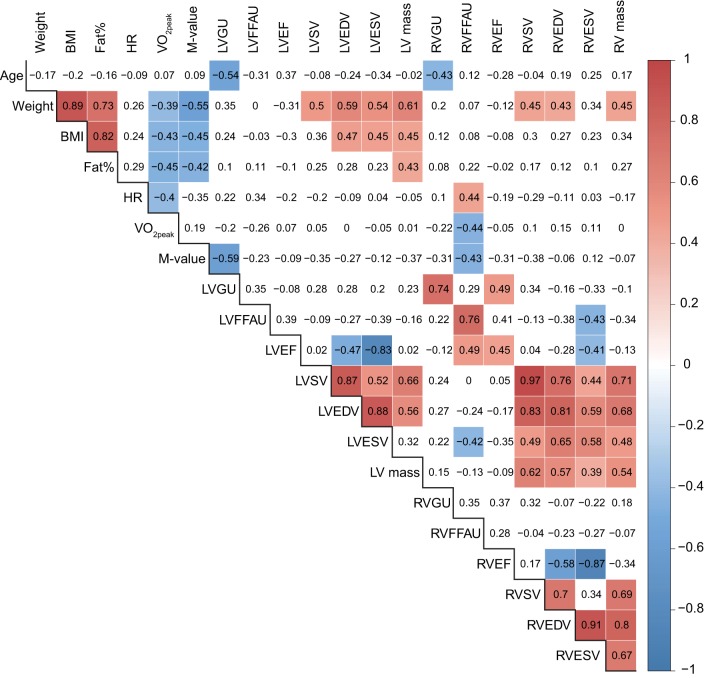
**Pairwise correlations for all measured variables**. The numbers indicate the correlation coefficient for a given pair of variables. Correlations that are statistically significant (*p* < 0.05) are highlighted with red (positive correlations) or blue (negative correlations) according to the color key on the right.

RVGU correlated negatively with age (*r* = −0.43, *p* = 0.034), whereas RVFFAU correlated positively with resting HR (*r* = 0.44, *p* = 0.031) and LVEF (*r* = 0.49, *p* = 0.018) and negatively with VO_2peak_ (*r* = −0.44, *p* = 0.030), *M*-value (*r* = −0.43, *p* = 0.045), and LVESV (*r* = −0.42, *p* = 0.046). LVGU was negatively correlated with age (*r* = −0.54, *p* = 0.005) and *M*-value (*r* = −0.59, *p* = 0.002) and positively with RVEF (*r* = 0.49, *p* = 0.015). As expected, correlations between LVGU and RVGU as well as LVFFAU and RVFFAU were high (*r* = 0.74, *p* < 0.001 for GU and *r* = 0.76, *p* < 0.001 for FFAU). The only statistically significant correlation for LVFFAU was with RVESV (*r* = −0.43, *p* = 0.039). The entire correlation matrix for all variables is shown in Figure 4.

### Lasso regression results

**Figure 5:**
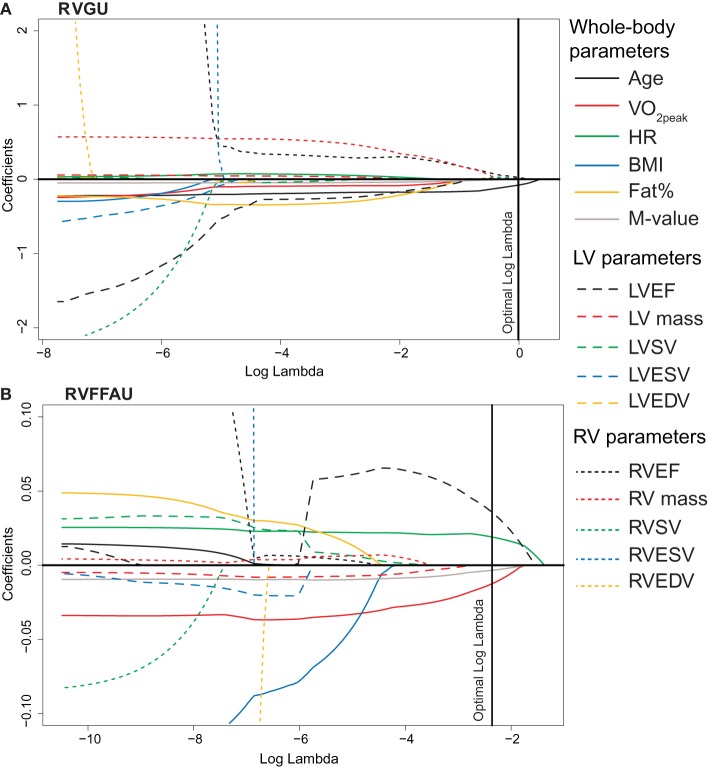
**The values of lasso regression coefficients as a function of logarithmic lambda for (A) RVGU and (B) RVFFAU**. The figures illustrate the order in which variables are dropped from or added to the model as parameter lambda is increased. Log lambda represents the penalizing factor for the sum of absolute values of coefficient. The left end of the curves illustrates the coefficients for standard linear regression without any penalty for the number of non-zero coefficients. When the penalizing factor lambda is increased, increasing number of non-significant variables is reduced to zero. The right end of the curves illustrates hypothetical maximal penalty where all the coefficients are reduced to zero. Hence, those variables that remain non-zero close to the right end of the curves are the most important predictors of RVGU or RVFFAU. The optimal log lambda value for each model is determined by repeated cross-validations. The reported lasso regression models in Table 3 are obtained from the non-zero coefficients at the optimal log lambda values. For example, model for RVGU includes variables age, RVEF, and RVSV, which are non-zero at optimal log lambda, whereas RV mass is the first term to be left out of the model (reduced to zero before optimal log lambda). The solid lines present whole-body parameters, dashed lines LV parameters, and dotted lines RV parameters according to the legend on the right.

**Figure 6:**
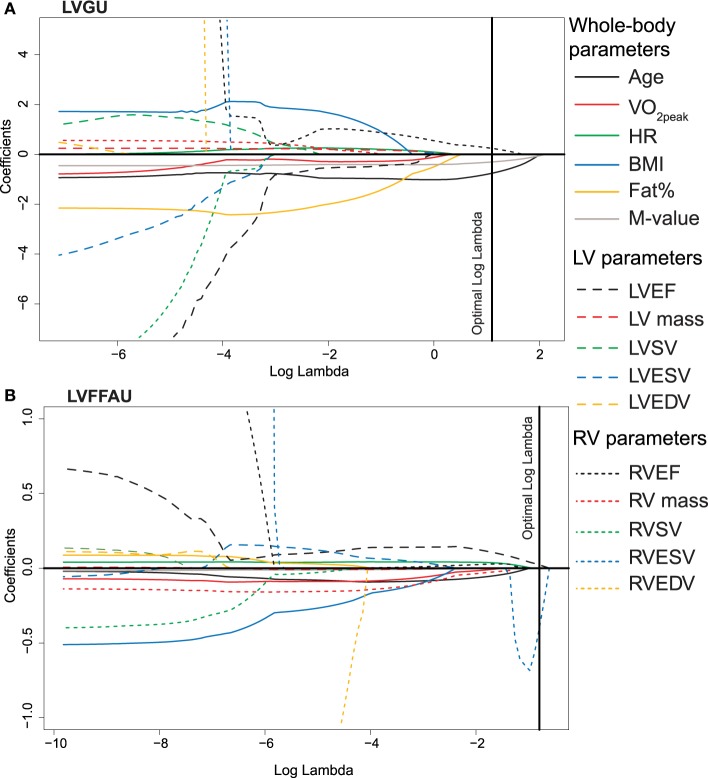
**The values of lasso regression coefficients as a function of logarithmic lambda for (A) LVGU and (B) LVFFAU**. See the details of the interpretation in Figure 5 legend.

Myocardial metabolism (RVGU, LVGU, RVFFAU, and LVFFAU) was modeled using lasso regression model. The variables included in each model were age, BMI, fat percent, resting HR, VO_2peak_, and *M*-value along with EF, SV, ESV, EDV, and mass of both ventricles. The obtained lasso regression equations are presented in Table 3. Considering glucose metabolism, age and RVEF were significant factors in both ventricles, with younger age and higher RVEF related to higher GU. For RVGU, RVSV was also a significant predictor, whereas *M*-value was a negative predictor of LVGU. In fatty acid metabolism, LVEF affected FFAU of both ventricles. In addition, RVFFAU was explained by resting HR, VO_2peak,_ and *M*-value while LVFFAU was related to RVESV. Figures 5, 6 illustrate the order (from left to right) in which the variables are dropped out or added to the lasso regression models for RV and LV metabolism, respectively.

## Discussion

**Figure 7:**
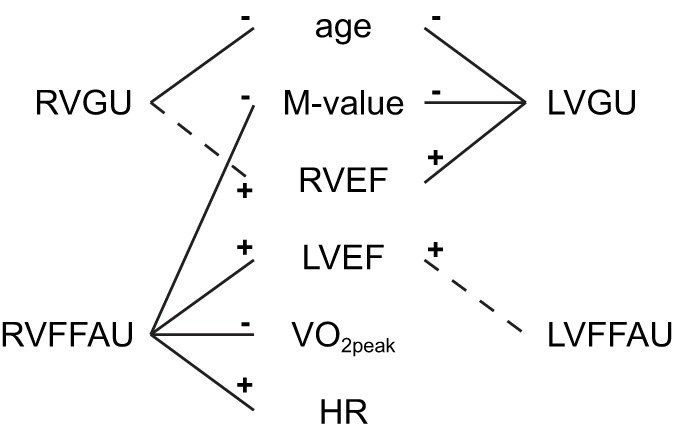
**Summary of the key findings of the present study on RV and LV metabolism**. Solid lines indicate associations that were confirmed by both lasso regression model and statistically significant pairwise correlation. Dashed lines present associations which were confirmed by lasso regression but pairwise correlations were not statistically significant. The associations summarized here were also supported by the heatmap (Figure 3). The signs indicate positive (+) and negative (−) associations.

The first aim of the present study was to investigate the determinants of the right ventricular glucose and free fatty acid metabolism in healthy middle-aged men. The main findings were that RVGU was predicted by age and RVEF. Interestingly, RVFFAU was related to VO_2peak_, resting HR, LVEF, and *M*-value which all can be considered as parameters related to physical fitness and health. The second aim of the study was to compare the predictors of RV and LV metabolism. As in RVGU, age and RVEF were also predictors of LVGU. However, the strongest and negative determinant of LVGU was *M*-value, which was not found for RVGU. Another difference between the ventricles was found in fatty acid metabolism. VO_2peak_ and resting HR were unique findings only for RVFFAU as these parameters were not among the strongest predictors for LVFFAU. Interestingly, ejection fraction was related to myocardial metabolism in such a way that RVEF was associated to GU while LVEF predicted FFAU of both ventricles. The key findings of the study are summarized in Figure 7.

### Predictors of RV metabolism

Based on the findings in this study, the strongest predictor for RVGU was age, implying that RVGU is decreased with increasing age among middle-aged men. Previous studies have shown differing results regarding the association between age and myocardial GU in LV: some report no correlation between these variables (de Groot et al., 2005; Jeong et al., 2013), while in other studies GU is decreased in older subjects (Israel et al., 2007), or even increased in older subjects (Kates et al., 2003). Also animal studies have shown divergent results in levels of age-related changes in myocardial glucose transporter isoform 4 (Cartee, 1993; Ozaki et al., 1996; Martineau et al., 1999). Possible explanations for different results are differences in imaging protocols (with or without insulin clamp), in quantitative analyses and also whether subjects were healthy or patients. However, our result on RVGU supports the statement that the age seems to affect the myocardial glucose metabolism and therefore age should be taken into account when performing measurements of RV myocardial glucose utilization (Peterson and Gropler, 2010).

Interestingly, the strongest predictors for RVFFAU were resting HR, VO_2peak_, LVEF, and *M*-value which all are related to physical fitness and health. Negative correlation was found between RVFFAU and VO_2peak_ and *M*-value and positive with resting HR, suggesting that better physical fitness is related to lower RVFFAU. Previous studies have compared the fatty acid utilization of the LV between lower and higher physically active twins (Hannukainen et al., 2007) and between endurance athletes and sedentary men (Turpeinen et al., 1996; Takala et al., 1999), and there were no difference in FFAU between the groups. Furthermore, fatty acid beta-oxidation index has not been found to be correlated with variables such as exercise capacity or the size of the heart (Turpeinen et al., 1996). However, the present study on RV metabolism suggests that it may be the RVFFAU that is associated with exercise capacity.

Clustering of the variables in heatmap (Figure 3) revealed that RV metabolism was related to ejection fraction so that RVGU was associated with RVEF while RVFFAU was surprisingly associated with LVEF. The connection between RVFFAU and LVEF was further supported by statistically significant correlation and by the lasso regression model, where LVEF was the most important factor for RVFFAU (Figures 4, 5). This connection suggests that there is an important interplay between LV and RV, in a way that LV function assists that of RV, which is reflected by its metabolism. While in the present study in healthy subjects, RVEF was increased with increasing RVGU, the opposite association has been found in patients with heart failure or PAH where increased RVGU was related to decrease of RVEF or other RV functional parameters derived from echocardiographic measurements (Can et al., 2011; Mielniczuk et al., 2011; Lundgrin et al., 2013; Yang et al., 2014). This may reflect different downstream metabolism in health and disease. In healthy heart, glucose is oxidized to ATP, whereas in PAH increased GU is associated to increasing portion of lactate-yielding glycolysis due to disease (Ryan et al., 2015). Thus, in healthy subjects increased RVEF is likely accompanied with increase in glucose oxidation while in diseased heart decreased RV function is associated with increased GU for glycolysis.

### Comparison of RV and LV metabolism

Common feature for both ventricles was the negative correlation between GU and age in middle-aged men. Also ejection fraction was associated with metabolism of both ventricles. An interesting finding was that RVEF is more closely associated with GU of both ventricles whereas LVEF was more tightly related to FFAU of both ventricles. This was supported by all the three analysis methods used in the study. Such ventricular interdependence has also been observed in patients with left heart failure, where RVEF is shown to be stronger predictor of survival than LVEF (Di Salvo et al., 1995; de Groote et al., 1998; Zornoff et al., 2002). The statistically significant correlations between RVFFAU and LVESV as well as between LVFFAU and RVESV further support the interdependence of the ventricles.

There were two main differences between RV and LV metabolism. Firstly, only LVGU was inversely related to whole-body glucose uptake. Similar finding was observed in a previous study on endurance athletes and sedentary subjects, where the athletes had enhanced whole-body glucose uptake but reduced LVGU compared to sedentary subjects (Nuutila et al., 1994). Interestingly, the present study on sedentary middle-aged men shows the same association without other confounding factors such as possible training-induced increase in whole-body insulin sensitivity or LV hypertrophy. The second difference was the association between RVFFAU and exercise capacity (VO_2peak_ and HR rest) which was not found for LVFFAU in these sedentary middle-aged men. Interestingly, the growing number of studies have indicated that it is the right ventricle that may in fact be more important during the exercise, and that its ejection fraction may be acutely compromised during strenuous endurance exercise most likely due to the relatively greater exercise-induced increase in RV work load (Trivax et al., 2010; La Gerche et al., 2011, 2012; Elliott and La Gerche, 2015). In line with this, the results obtained in the present study suggest that particularly the right ventricular metabolism may be more related to exercise capacity than that of the left ventricle.

### Study limitations

Although lasso regression models for GU and FFAU were constructed, the models are based on rather small study population (*n* = 28) and no separate validation data was available. However, the results obtained in the presents study were confirmed by several analyses methods (pairwise correlations, visualization by heatmap, and lasso regression model) which supported each other, increasing the validation of the findings.

GU and FFAU were measured in different metabolic conditions (GU during insulin clamp and FFAU in the fasted state) in this study. Therefore, the GU and FFAU rates should not be directly compared. This selection was done to mimic the conditions when they both are highest in normal daily life. That is, after a meal (mimicked with insulin infusion during glucose-insulin clamp) when GU is highest and in fasted state when FFAU is highest.

## Conclusions

The glucose utilization of both ventricles was lower in older subjects. Therefore, age should be taken into account when measuring myocardial glucose uptake. Interestingly, myocardial metabolism was found to be associated with ejection fraction and even so that RVEF was more strongly associated with GU and LVEF to FFAU of both ventricles, a result showing ventricular interdependence. The main differences between the ventricles were that the whole-body glucose uptake was related only to LVGU, whereas only RVFFAU was related to maximal exercise capacity and resting heart rate in sedentary middle-aged men. While the physiologic relevance or mechanism for the findings of the present study remains unclear, this study highlights the importance of further study designed specifically on RV as the results on LV metabolism and physiology may not be directly applicable to RV.

## Author contributions

All authors were involved in the planning of the study. JE, IH, KV, JP, JH, and KK collected the data. MH, TL, JE, and EL analyzed the data. MH and KK drafted the manuscript. All authors revised the manuscript. All authors have approved the final submitted form of the manuscript.

## Funding

This study was conducted within the Centre of Excellence in Cardiovascular and Metabolic Research, supported by the Academy of Finland, the University of Turku, Turku University Hospital, and Åbo Akademi University. The study was financially supported by the Academy of Finland (grants 251399, 251572, 256470, 281440, and 283319), the Ministry of Education of the State of Finland, the Paavo Nurmi Foundation, the Finnish Cultural Foundation, the Novo Nordisk Foundation, the European Foundation for the Study of Diabetes, the Hospital District of Southwest Finland, the Orion Research Foundation, the Finnish Cardiovascular Foundation, the Finnish Diabetes Foundation, and the Yrjö Jahnsson Foundation.

### Conflict of interest statement

The authors declare that the research was conducted in the absence of any commercial or financial relationships that could be construed as a potential conflict of interest.

## Abbreviations

BMI: body mass index
CMR: cardiac magnetic resonance
EDV: end-diastolic volume
EF: ejection fraction
ESV: end-systolic volume
FDG: 2-deoxy-2-(^18^F)fluoro-D-glucose
FFAU: free fatty acid uptake
FTHA: 14(*R,S*)-(^18^F)fluoro-6-thia-heptadecanoic acid
GU: glucose uptake
HR: heart rate
LV: left ventricle/ventricular
*M*-value: whole-body insulin-stimulated glucose uptake
PET: positron emission tomography
RV: right ventricle/ventricular
SV: stroke volume
VO_2peak_: peak oxygen consumption.
